# Infectious diseases, NCDs, child and reproductive health

**DOI:** 10.4314/ahs.v26i2.1

**Published:** 2026-06

**Authors:** James K Tumwine

This June Issue of *African Health Sciences* comes in the wake of an Ebola outbreak in the Democratic Republic of the Congo (DRC), right in the heart of Africa.^1^ It reminds us of the DRC as the origin of Ebola, and as it grapples with this epidemic, we are sadly reminded of the fact that Ebola is a name of a river in the same country where the first Ebola outbreak was described. Somewhat ironically, the DRC has been linked with yet another neglected infectious disease – monkey pox.^2^

Even though these epidemics are deadly with high fatality, Africa is, also, grappling with other infectious diseases that need world attention: malaria and tuberculosis.

It is with this in mind that we have selected for you several papers on tuberculosis.

These include a paper on hypocholesterolemia and severity of tuberculosis^3^; and adherence to TB treatment.^4^ Keeping the theme of adherence, scientists from Bulawayo in Zimbabwe have written for us a paper on Non-Adherence and first line Antiretroviral Therapy (ART) failure among people living with HIV on ART.^5^

From North Africa, we have a paper on Enterococcus faecium vancomycin and linezolid resistance.^6^ The treatise on infectious diseases ends with work from Indonesia on incomplete immunization coverage and its associated factors among children aged 1-5 years.^7^

Next we have results of a pilot study on diversity of ß-globin gene cluster haplotypes in Moroccan sickle cell disease patients.^8^ Keeping with the sickle disease theme, we have a study on diagnostic relevance of osteocalcin and bone alkaline phosphatase as markers of bone turnover in Sickle Cell Anaemia.^9^ Now to diabetes. Here, we have a paper on prediabetes and diabetes among patients presenting with acute stroke or acute coronary syndrome at a tertiary Regional Referral Hospital in Kenya.^10^ This is followed by a paper from Ethiopia on non-adherence to diabetes treatment among patients in a referral hospital in Hawassa.^11^ Then follows a list of papers with a Nigerian touch: patients' perceptions of the role of community pharmacists in the prevention and control of cardiovascular diseases;^12^ and a scoping review of chronic kidney disease awareness, prevention and policy gaps with implications for the Nigerian Navy.^13^ Treatment methods and recurrence rates in primary spontaneous pneumothorax: a 10-year retrospective study.^14^

Now to reproductive health issues led by a Ugandan paper on risk factors for pregnancy among rural and urban-dwelling adolescents in a high adolescent-pregnancy setting; a case control study.^15^ Perinatal depression and maternal-infant bonding disorders in mothers of full-term normal infants.^16^ It is followed by one on relationship between coffee consumption and vasomotor symptom severity in Turkish postmenopausal women.^17^ Then comes understanding barriers to treatment regimen adherence in Pakistani women with polycystic ovary syndrome: a cross sectional study.^18^ Barriers to quality oral health care among elderly (geriatric) patients in Oyo State.^19^ Knowledge and risk perception of pesticide exposure among smallholder farmers of Sembabule district, Uganda.^20^ Optometry clinical training and practice at the public health facilities in South Africa: a documentation review.^21^

Relax and enjoy this very interesting selection.
